# The temporal priority principle: at what age does this develop?

**DOI:** 10.3389/fpsyg.2013.00178

**Published:** 2013-05-08

**Authors:** Michelle L. Rankin, Teresa McCormack

**Affiliations:** School of Psychology, Queen’s University BelfastBelfast, Country Antrim, Northern Ireland

**Keywords:** causal reasoning, time, development

## Abstract

The temporal priority principle states that all causes must precede their effects. It is widely assumed that children’s causal reasoning is guided by this principle from early in development. However, the empirical studies that have examined children’s use of the principle, most of which were conducted some decades ago, in fact show inconsistent findings. Some researchers have argued that 3-year-olds reliably use this principle, whereas others have suggested that it is not until 5 years that children properly grasp the inviolability of the principle. To examine this issue, 100 children, 50 three-year-olds, and 50 four-year-olds, took part in a study in which they had to judge which of two causes yielded an effect. In the task, children saw one event (A), an effect (E), and then another event (B). The events A and B involved the rolling of balls down runways, and the effect E was a Jack-in-a-box popping up. The extent to which E left a visible trace was also varied, because comparisons across previous studies suggested that this may affect performance. As a group, 3- and 4-year-olds performed at above-chance levels, but performance improved with age. The nature of the effect did not have a significant impact on performance. Although some previous studies suggested that 3-year-olds may be more likely to choose B rather than A as a cause due to a recency effect, we found no evidence of this pattern of performance in the younger group. Potential explanations of the age-related improvement in performance are discussed.

## INTRODUCTION

Much of the recent research on children’s causal learning has focused on their ability to use statistical information to make causal inferences, with such learning modeled using the causal Bayes net approach (e.g., Gopnik and Schulz, 2004; Gopnik et al., 2004; Sobel et al., 2004; Gopnik, 2012; see Lu et al., 2008; Griffiths and Tenenbaum, 2009 for more recent Bayesian approaches to causal learning). Researchers in this tradition have typically emphasized the good performance of young children in causal learning tasks (see Gopnik and Schulz, 2004; Gopnik, 2012 for review), suggesting developmental continuity in causal cognition. However, important questions still remain about whether there may be qualitative differences between younger and older children’s causal abilities, with such differences being a theme of the body of research on children’s causal cognition that pre-dated the Bayesian approach (e.g., Shultz and Mendelson, 1975; Bullock, 1985; Corrigan and Denton, 1996; Schlottmann, 1999). In this paper, we focus specifically on whether there are developmental changes in children’s grasp of the principle that causes must precede effects.

The temporal priority principle in causal reasoning states that causes always come before their effects in time, even though there may appear to be some circumstances in which cause and effect appear perfectly contemporaneous (for example, when we press a button on a TV remote, or turn the volume up or down on the radio). Although some philosophers have argued for the possibility of backward causation (Dummett, 1954), in everyday life there would never be a circumstance in which causal attributions would knowingly be in breach of the temporal priority principle, i.e., adults would never choose the succeeding event as the cause of a given effect. As this principle, and causal cognition more generally, is fundamental for success in the world which surrounds us, it is important that we determine the age at which the inviolability of the principle is properly appreciated.

Most of the studies that examined children’s use of the temporal priority principle did so in the 1970s and 1980s (Shultz and Mendelson, 1975; Kun, 1978; Bullock and Gelman, 1979; Sophian and Huber, 1984). However, in recent years there has been a resurgence of interest in investigating temporal cues to causation in both adults’ and children’s judgments (Buehner and May, 2004; Lagnado and Sloman, 2004, 2006; Buehner, 2005; Buehner and McGregor, 2006; Burns and McCormack, 2009; Greville and Buehner, 2010; Frosch et al., 2012). Some of these studies have addressed whether children and adults will infer the structure of events in a causal system, based on the temporal pattern in which events occur (Lagnado and Sloman, 2004, 2006; Burns and McCormack, 2009; Frosch et al., 2012), whereas others have examined how the temporal contiguity of an event and an outcome affect causal strength judgments (Buehner and May, 2004; Buehner, 2005; Buehner and McGregor, 2006). In both of these sorts of studies, it is assumed that participants’ causal judgments will respect the temporal priority principle. However, we can query whether such an assumption is appropriate with respect to young children.

As we have mentioned, most of the empirical research into children’s use of the temporal priority principle was conducted some decades ago, conclusions were drawn on the few studies conducted, and then the issue was then evidently laid to rest. Based on these studies, review papers such as that of Bullock et al. (1982) have drawn clear conclusions about the age at which young children respect the principle when making causal judgments (Bullock et al., 1982; Burns and McCormack, 2009). The study that is most frequently referred to is that of Bullock and Gelman (1979), which found that children as young as 3 years of age chose a temporally prior event as the cause of an effect more often than would be expected by chance. Based on their findings, researchers will typically assume that 3-year-olds are capable of understanding the fact that causes always precede effects in time (Bullock et al., 1982). However, on closer inspection of studies investigating the temporal priority principle, it is apparent that despite the principle’s fundamental nature, the empirical evidence regarding children’s ability to adhere to this principle in their causal judgments is mixed (Shultz and Mendelson, 1975; Kuhn and Phelps, 1976; Kun, 1978; Bullock and Gelman, 1979; Shultz et al., 1986). Indeed, we would argue that on the basis of the current evidence available, it is not clear at what age children properly appreciate the principle when making causal judgments.

One possible reason for the mixed pattern of findings is that the most common studies referred to in the literature have included relatively small sample sizes. For example, in Bullock and Gelman’s (1979) study, there were only 16 children in each age-group and in Shultz and Mendelson’s (1975) study there were only 18 in each age-group. Given the potential variability in performance by young children, differences in findings between studies could reflect characteristics of children in the samples. Arguably, these sample sizes are potentially not sufficient to provide a true or detailed representation of performance of the age-groups examined, and we included much larger sample sizes in the current study. Moreover, it is clear that, looking across the studies, researchers who have investigated children’s use of the temporal priority principle have sometimes used very different methodologies. Most notably, some researchers asked children questions about pictures of familiar event sequences, whereas others used novel mechanical events and asked children to make causal attributions. This makes it difficult to directly compare each study, and draw firm conclusions based on their findings.

One technique used to examine children’s use of the temporal priority principle involves two events, event A and event B, with children being required to choose the event that caused the other event to occur (Sophian and Huber, 1984; Shultz et al., 1986). Shultz et al. (1986) found that 3- to 4-year-olds failed to do this correctly when making causal judgments. However, one problem with the methodology in Shultz et al.’s (1986) study may be that, for some of the trials, A caused B to occur, while in other trials the causal order was reversed. Thus, there was not one standard cause and one standard effect. This could have been confusing for young children, and may have led to random responding. In many everyday scenarios, the roles of cause and effect cannot be reversed; for example, pressing a switch will cause the light to turn on but the light turning on does not cause the switch to be pressed. This suggests that the technique may not be the most appropriate one to use when investigating temporal priority.

A second technique involved presenting children with pictures depicting sequences of events, and children were then asked questions about causality (Kun, 1978; Das Gupta and Bryant, 1989). For example, Kun (1978) presented 4.5-, 6-, 7-, and 8-year-olds with 10 sets of three picture cards in the form “A caused B caused C,” where A was the antecedent of B and C was the consequent of B. For instance, one set of cards depicted a child pulling a dog’s tail (A); the dog biting the child (B); the child crying (C). Children were then asked three questions: what happened next (pointing at B), why did B happen, and a non-sense question. They simply had to point to the picture that answered the question. Kun (1978) found that children as young as 4 years answered these questions in a way that suggested that they understood the temporal priority principle: that is, they were able to choose the correct antecedent and consequent of B. A potential issue with this experiment is that many of the sequences presented to the children may have been familiar to them due to previous experiences and exposure to the events. Hence, the children may have already developed schemas about the events and the consequences of many actions, which might affect how they respond in this experiment.

A third technique that overcomes the problems identified with both of the other techniques, and is the one which was adopted by the current study, is what we have termed the A–E–B paradigm. This technique has been used in a number of previous studies (Shultz and Mendelson, 1975; Bullock and Gelman, 1979; Bullock et al., 1982; Sophian and Huber, 1984). It requires children to draw inferences about which of two possible events (A or B) caused an outcome. For example, children are shown a potential cause A, followed by effect E, and then potential cause B. In this case, the correct response is to choose A as the cause as it precedes effect E. Advantages of this paradigm are that, first, the events and sequences used in the paradigm can be chosen to be novel to children. Thus, it removes the opportunity for children to draw upon their knowledge of the causal power of familiar events. Moreover, the roles of cause and effect are never switched, unlike in the studies of Shultz et al. (1986) and Sophian and Huber (1984, Experiment 1). Finally, the paradigm draws very little on verbal abilities and so it cannot be argued that children’s comprehension and verbal ability are confounding their performance.

Bullock and Gelman (1979) and Shultz and Mendelson (1975) adopted this technique in their studies. Shultz and Mendelson (1975) used three different pieces of equipment in their study, one of which we will focus on here. This piece of equipment was a wooden box with two holes on top at either end, with events A and B being the dropping of marbles into the holes and the effect being a bell ringing. Bullock and Gelman (1979) only used one piece of apparatus in their study, but it was very similar to the piece of equipment used by Shultz and Mendelson (1975). Bullock and Gelman’s (1979) apparatus also involved a wooden box with two holes on top of the box, at either end. The experimenter would drop two balls into the holes (A or B) and children were then required to decide which ball caused the effect (E). The sequence always took the form A–E–B or B–E–A. Although in Shultz and Mendelson’s (1975) study the effect (E) was a bell which rang inside the box, in Bullock and Gelman’s (1979) study the effect was a teddy bear which popped up, giving a “Jack-in-the-box” effect.

Shultz and Mendelson (1975) found that 6- to 7-year-olds and 9- to 11-year-olds chose the preceding event as the cause whereas 3- to 4-year-olds were as likely to attribute the cause to the following as well as the preceding event. In fact, Shultz and Mendelson (1975) compared the performance of younger 3-year-olds (aged 3 years 0 months to 3 years 7 months) with the performance of older 3-year-olds (3 years 8 months to 3 years 11 months) and, interestingly, found that the younger 3-year-olds tended to erroneously choose the event that occurred most recently as the cause more often than would be expected by chance. This in itself raises an issue: why did younger 3-year-olds choose the most recent event as the cause, rather than simply responding at random? Shultz and Mendelson (1975) suggested that if young children do not understand the temporal order of the cause and effect process they will choose the event that is most salient to them, which in this case would be the most recent event. In contrast, Bullock and Gelman (1979) found that children as young as 3 years of age consistently chose the preceding event as the cause. Thus, unlike the conclusion drawn by Shultz and Mendelson (1975); Bullock and Gelman (1979) argued that their results suggest that children as young as 3 years old can and do rely on temporal ordering when making causal judgments (Bullock and Gelman, 1979). Also, like adults, they understand that temporal priority dominates over all other cues, for example, spatial contiguity (Bullock et al., 1982).

Considering both these studies used similar apparatus and a similar methodology, it is surprising that they found such contradictory results. Moreover, from these results, very different conclusions have been drawn about the age when children fully comply with the temporal priority principle. This prompted the current study to investigate this issue further, in an attempt to determine not only the age at which young children comply with the temporal priority principle, but also to discover why Bullock and Gelman (1979) and Shultz and Mendelson (1975) found conflicting results.

There are small methodological differences that could potentially provide an explanation for the differences in the results. One notable difference is in relation to how the experimenters obtained responses from children. Shultz and Mendelson (1975) asked children a series of quite complex questions whereas Bullock and Gelman (1979) opted for a more simple response method, using questions that young children may have been more likely to understand. In addition to this, although the apparatus used in both studies included runways, it was only in Bullock and Gelman’s (1979) apparatus that the runways were actually visible to children. Thus, there was a difference in spatiotemporal and mechanism information between the two studies

There were two other methodological differences that may have potentially affected the amount of attention that children paid to the B event in the sequence. In Bullock and Gelman’s (1979) paper, they stated that event B occurred “coincident with the start of the Jack’s action,” i.e., that there was no delay between event E and B, whereas in Shultz and Mendelson’s (1975) study, there was an obvious delay between event E and event B. Moreover, the studies differed in the extent to which the effect (E) left a visible trace once it occurred in the sequence of events. In Shultz and Mendelson’s (1975) study, the effect was a bell, which rang. There was no visible trace of the effect, and the bell had stopped ringing before the second potential cause occurred. By contrast, in Bullock and Gelman’s (1979) study, once the “Jack” popped up, it remained up even when the second ball was released into the hole. It could be argued that these variations – in the delay between B and E and in the persistence of the causal consequence – may potentially explain the difference in results obtained across these studies. In particular, these differences may have had an influence on children’s attention while they observed the sequence of events. In Bullock and Gelman’s (1979) study, once the “Jack” popped up and remained up, attention may have been so focused on the “Jack” that children may not have witnessed the second ball being released into the hole. Therefore, when asked to choose the ball that made the “Jack” pop up, they may have chosen the first ball because this was the only ball that they had attended to. Conversely, as mentioned earlier, in Shultz and Mendelson’s (1975) study the bell stopped ringing before the second ball was released into the hole and so this may have allowed children to concentrate on the second ball and increased the likelihood it was chosen as being causally relevant to the effect. Alternatively, it could be argued that the persistent visibility of the “Jack,” and its intrinsic interest to children, increased the salience of the effect which in turn may have made it easier for the children to correctly recall the order of events, and as a result, choose the correct event as the cause.

It seems plausible that these variations in Shultz and Mendelson’s (1975) and Bullock and Gelman’s (1979) methodologies could explain the difference in results, and that they could be explored in an attempt to explain the variation in results. However, more importantly, with regards the issues regarding existing studies that were raised earlier, it is appropriate to return to the question of when children reliably appreciate the temporal priority principle in their causal reasoning. Thus the primary aim of the current study was to attempt to determine the age at which young children adopt the principle of temporal priority when making causal judgments. Additionally we were interested in whether Shultz and Mendelson (1975) were correct to argue that younger 3-year-olds will tend to answer systematically incorrectly in this type of task, by showing a recency effect in their causal judgments. The current study used a large sample of children aged 3 and 4 years, with participants assigned to one of two conditions. In one condition, the “Jack up” condition, the effect involved a teddy popping up and staying in view, whereas in the other condition, the “Jack down” condition, the teddy bear popped up and then went down again. The key difference between these conditions was in whether there was a persistent visually available effect when the second possible cause occurred.

## MATERIALS AND METHODS

### PARTICIPANTS

One hundred children took part in the study, fifty 3-year-olds (*M* = 43 months; range = 36–47 months) and fifty 4-year-olds (*M* = 53 months; range = 48–59 months). There were 64 females and 36 males in total. Half of each age-group was randomly assigned to one of two experimental conditions. Children were recruited in local schools and preschools. All the children were tested individually in the child’s school or preschool and each child received a sticker for taking part.

### APPARATUS

**FIGURE 1:**
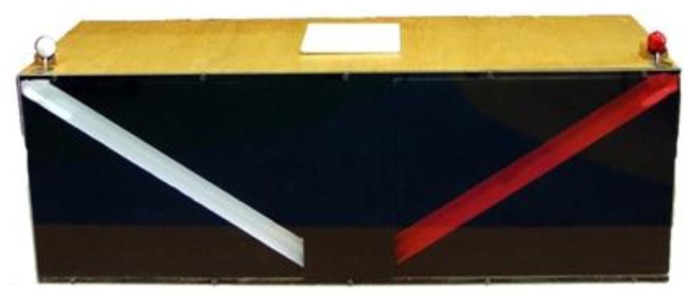
**Diagram of the front view of the apparatus**.

The apparatus consisted of a wooden box, 70 cm long, 21.5 cm high, and 23 cm wide. On the left and right sides of the box were plexiglass windows showing two runways inside the box. One runway was painted red (the location of event A) and the other runway was painted white (the location of event B) so children could discriminate between the two. The runways were 30 cm long, began at openings on the top outer corners of the box and dropped at a 30° angle toward the center of the box. The runways were a mirror image of each other (see **Figure 1**).

White and red wooden balls (2 cm in diameter) were placed onto holders situated at the edge of the openings of the box. A button could be pressed at each holder to release the ball into the opening, and begin the sequence of events. When the balls were released they rolled down the runways, disappeared from view, and silently rolled into a compartment at the back of the box that was easily accessible to the experimenter and out of sight from the child.

On the top middle section of the apparatus was a 10 cm × 10 cm opening to which a lid was attached, and a teddy bear Jack-in-the-box (event E) was located beneath this lid and could pop up from it. Even though the first event was manually instigated by the experimenter, unbeknownst to children, the teddy bear popping up and release of the second ball was driven by hidden motors and built-in timers. This allowed for maximal control over the sequence of events and their timing. The timing between A and E, E and B (in an A–E–B sequence), and B and E, and E and A (in a B–E–A sequence) was fixed at 1.5 s between each event. By means of a digital video recording, we estimated that the amount of time a ball was visible as it rolled down a runway was 0.38 s. Thus, once the ball was released into the runway (event A), it took 0.38 s for it to roll down the runway and disappear from view. Then after another 1.12 s, the teddy bear popped up (event E). Once the brief Jack-in-the-box event had finished, after another 1.5 s, ball B was released into the runway (event B), and 0.38 s later, ball B disappeared from view.

### DESIGN

There were two experimental conditions, labeled “Jack up” condition and “Jack down” condition. The design was between subjects with children in each age-group randomly assigned to one of the two experimental conditions. In both conditions, the sequence of events was initiated by the experimenter pushing the button that released the ball into the hole. In the “Jack up” condition, after 1.5 s, the Jack popped up and remained up (effect E), and after another 1.5 s, the second ball was released into the hole. In the “Jack down” condition, after 1.5 s, the Jack popped up and then disappeared back down again under the lid. After another 1.5 s the second ball was released into the hole.

### PROCEDURE

At the beginning of the testing session, children were invited to take a seat facing the box and were told that they were going to play a game with the experimenter. They were introduced to the “special box,” and then asked to name the colors of the runways and the wooden balls. The children were then asked to watch very carefully as the experimenter placed a wooden ball onto the holder and released it into one of the runways. They watched the experimenter initiate four training demonstrations (A–E, A–E, B–E, B–E, or B–E, B–E, A–E, A–E, with order counterbalanced across the groups) after which they were asked two closed, forced choice questions. One was about ball A and the other about ball B: “Did the white ball make the teddy bear pop up?” and “Did the red ball make the teddy bear pop up?” This training phase was included in order to demonstrate that either ball could potentially cause the “Jack” to pop up. Thus, correct judgments at test had to be based solely on the temporal order cues available to children. If children answered these questions correctly the experimenter moved onto the test trials, but if children answered incorrectly the experimenter repeated the training phase again.

During the test trials, children saw four sequences of events. They observed one ball being released into the runway, disappear, the teddy bear popping up and then the second ball being released into the runway. Although it looked to children that the experimenter released the ball, because the experimenter’s hand was held directly behind the ball on its holder, in fact it was released by a timing mechanism. Children saw the A–E–B sequence twice and the B–E–A sequence twice. Thus, twice the correct answer was to choose the red ball, and twice the correct answer was to choose the white ball. The order in which the trials were presented was randomized for each child. After each trial children were asked to choose the ball that “made the teddy bear pop up.” No feedback was given. Once participation was complete, children were thanked for their participation, given a sticker and were then returned to their classroom.

## RESULTS

**FIGURE 2:**
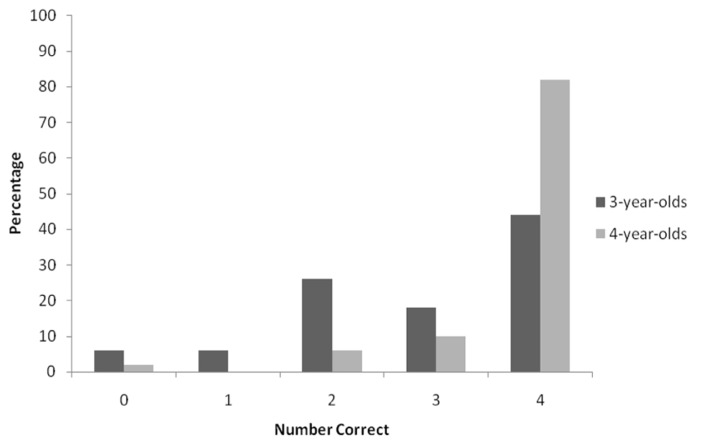
**Frequencies of correct responses by 3- and 4-year-olds across the four experimental trials collapsed across both conditions**.

**Table 1** shows the mean number of times children chose the temporally prior event in each of the two conditions. It is clear that 4-year-olds performed better in both conditions. The table also shows that, for both age-groups, there appears to be very little difference between how children performed in each of the two conditions. **Figure 2** shows the frequencies of correct responses of 3- and 4-year-olds collapsed across both conditions. The majority of the 4-year-olds chose the temporally prior event in each of the four trials: 41 performed perfectly compared to only 22 three-year-olds. An analysis of variance (ANOVA) was conducted with between groups factors of condition and age-group. The results revealed no significant main effect of condition and the interaction between age-group and condition was also not significant, both *F*s < 1. There was however a significant main effect of age-group *F*(1,99) = 15.90; *p* < 0.001. This result indicates that 4-year-olds chose the temporally prior event significantly more often than 3-year-olds. However, although 3-year-olds did not perform as well as 4-year-olds, a one-sample *t*-test (with a test value of 2, because children completed four trials) revealed that 3-year-olds’ performance was significantly above chance, *t* = 5.09; *df* = 49; *p* < 0.001. As would be expected, a one-sample *t*-test revealed that 4-year-olds’ performance was also significantly above chance, *t* = 15.76; *df* = 49; *p* < 0.001.

**Table 1:** Number of times children chose the temporally prior event in each of the two conditions.

**Age-group**	**Jack-up**		**Jack-down**	
	**Mean**	**SD**	**Mean**	**SD**
3 years	2.84	1.31	2.92	1.15
4 years	3.64	0.91	3.76	0.60

*Scores can range from 0 to 4.*

*There were 50 children in each age-group.*

In light of Shultz and Mendelson’s (1975) findings it was decided to investigate 3-year-olds’ performance in more detail to examine if the younger 3-year-olds were likely to attribute the cause to the event that followed the effect rather than the event that preceded the effect. For this analysis, the responses of the 23 youngest 3-year-olds (3 years, 1 month to 3 years, 7 months, *M*: 3 years, 4 months) were compared with those of the 27 oldest 3-year-olds (3 years, 8 months to 3 years, 11 months, *M*: 3 years, 9 months). The mean number of correct responses for the younger group was 2.78 in the Jack up condition and 2.64 in the Jack down condition; means for the older group were 2.88 and 3.27, respectively. A two-way ANOVA with factors of age (young versus old 3-year-olds) and condition revealed no significant main effects or interactions, all *Fs* < 1. Therefore, there was no significant difference in how the younger 3-year-olds responded compared to how the older 3-year-olds responded. In addition, we examined the age of children who achieved each of the five possible scores; **Table 2** shows the mean and range of these ages. It can be seen from the table that the children who were likely to erroneously choose the most recent event as the cause (those scoring 0 or 1) were not notably younger than those who simply responded at chance levels (scoring 2), and indeed that the groups of children with the higher scores included some of the youngest children. Thus, there was no indication in our data of a developmental shift from below-chance to above-chance performance. In fact, **Figure 2** clearly shows that within the group as a whole, relatively few 3-year-olds systematically chose the most recent event as the cause.

**Table 2:** Age in months of children achieving each score.

	**Number correct**				
	**0**	**1**	**2**	**3**	**4**
Jack up	*N* = 3	*N* = 2	*N* = 6	*N* = 8	*N* = 31
Mean age	46.33	44.50	45.67	48.50	50.35
SD	4.51	3.54	2.73	6.48	5.82
Range	42–51	42–47	41–49	42–59	36–59
Jack down	*N* = 1	*N* = 1	*N* = 10	*N* = 6	*N* = 32
Mean age	47	39	41.16	46.17	49.81
SD	–	–	6.53	6.68	5.42
Range	–	–	36–57	39–58	39–59

## DISCUSSION

Adopting the A–E–B paradigm, and using similar apparatus to that used in two influential studies, we attempted to discover whether the causal judgments of both 3- and 4-year-olds would reliably reflect the principle that causes must always precede their effects in time. The key findings were that while children in both age-groups judge that a preceding rather than a succeeding event was the cause of an effect more often than would be expected by chance, there was a significant developmental improvement in the numbers of correct responses between the two ages. In the study, we also manipulated between two conditions whether or not the outcome was one that was visually present at the time at which the succeeding event occurred, because we had hypothesized that this may affect the likelihood that children would perform well on this task. However, there was no significant difference in levels of performance across the conditions.

The performance of the 4-year-olds was very good in both conditions, with children’s judgments rarely defying the temporal priority principle. Three-years-olds’ judgments were much less likely to be correct. Although as a group they performed significantly above chance, **Figure 2** shows something resembling bimodal performance in this age-group, with around a quarter of 3-year-olds performing at chance levels (two correct) and just below a half of the group getting all questions correct. The findings suggest that while some 3-year-olds seem to have firmly grasped the temporal priority principle, others are not yet reliably incorporating this principle in their causal judgments. Following Shultz and Mendelson’s (1975) analyses of younger versus older 3-year-olds’ performance, we examined whether the performance of our 3-year-olds differed depending on their age. However, there was no difference between how younger 3-year-olds performed compared to older 3-year-olds. Moreover, there was no tendency for young 3-year-olds to choose the succeeding event as the cause, and thus, these findings are not consistent with those of Shultz and Mendelson (1975).

In fact, our findings are not completely consistent with those of either of the early studies that our paradigm is based on. Unlike Bullock and Gelman (1979), we found that 4-year-olds performed significantly better than 3-year-olds. However, unlike Shultz and Mendelson (1975), we found that the younger group as a whole performed at above-chance levels. As it was found that the difference in the visibility of the “Jack” did not influence how children responded, this suggestion can be discarded as an explanation for the reason why these two previous studies found such contradictory results. It may be the case that, as discussed earlier, there are other procedural differences between the two previous studies that could potentially account for the variance in their results. Moreover, it should also be highlighted that we found marked individual differences in children’s performance levels in our 3-year-old group. Thus, the differences in findings between the previous studies could also reflect the varying ability levels of young children in the relatively small samples they tested.

It should be noted that the current study opted for a procedure that differed from both Bullock and Gelman’s (1979) and Shultz and Mendelson’s (1975) procedures (i.e., neither condition in our study was an exact replication of either of their methodologies). We used simple questions like those of Bullock and Gelman’s (1979), as we felt that Shultz and Mendelson’s (1975) questioning procedure was too complex. However, our procedure did differ from that of Bullock and Gelman’s (1979) in that we left an obvious delay between event E and event B, in order to match the delays between A and E and E and B (assuming an AEB sequence). We note that in our procedure, even though these delays were identical, once B occurred the ball took a further 0.38 s to pass down the runway and reach the Jack. Thus, the causally relevant component of the B event (the ball reaching the Jack) was more temporally separated from the effect than that of the A event. We deliberately introduced a clear delay between E and B so that children’s attention would not be divided between E and B (which occurred in different spatial locations). However, it might be argued that the consequence of this – the different temporal contiguity between E and the causally relevant components of the A and B events – may have biased participants in favor of choosing A. One way to avoid this problem would be to cover the runways up completely, so that children simply see the balls being dropped into the box, and do not see any additional visuo-spatial information. In fact, we have carried out such a study (Rankin and McCormack, unpublished), and found extremely similar results to those reported here.

An important question that remains is why the ability to consistently apply the temporal priority principle improves significantly between 3 and 4 years. We can distinguish between at least two possible explanations of the age effect: that changes reflect improvements in information processing efficiency (a processing explanation), or that changes reflect a new appreciation and understanding of the temporal priority principle itself (a reasoning explanation). With regard to the former explanation, a likely candidate process may be that of memory. Indeed, it has been argued that memory limitations may contribute to the difference in performance between 3- and 4-year-olds (Shultz and Mendelson, 1975; Kun, 1978; Koslowski and Masnick, 2002). It could be the case that 3-year-olds cannot remember the order of the event sequence in some trials, and, although they may be trying to use the temporal priority principle in their judgments, they are more likely to make errors than 4-year-olds because they mis-remember the sequence.

It seems likely that remembering event order and using this information to make an inference places demands on young children’s working memory resources. While the development of working memory has been extensively explored across childhood, we are only aware of a single study that has directly examined how it may affect children’s causal judgments. McCormack et al. (2013) measured working memory abilities alongside 4- to 7-year-old children’s causal learning in the context of a quite different causal learning task (one examining the cue competition effect of blocking). They found that children’s performance on the task was predicted by their working memory abilities over and above chronological age and verbal ability. Thus, there is some evidence that even relatively basic causal judgments might be affected by children’s working memory skills. We are currently exploring whether this is also the case in a causal task similar to that used in the present study.

It may also be the case that 3-year-olds’ understanding of causal principles is not as advanced as 4-year-olds’. This suggestion draws upon research that has investigated children’s use of temporal cues to make causal inferences (McCormack and Hoerl, 2005, 2007; McColgan and McCormack, 2008). The results from these studies suggest that children’s may be able to represent or remember the temporal order of events before they properly understand of the causal significance of this order. For example, McCormack and Hoerl (2007) conducted a study in which children were required to judge the outcome of a sequence of events based on the order in which events A and B occurred. Children were introduced to two dolls, John and Peter, and they were told that the dolls take turns to do things but one doll (e.g., John) always goes first and the other doll (e.g., Peter) always goes last. Children were then told that the dolls were going to go into a room to brush their hair. The experimenter closed the door of the room so that the children could not see what was happening, although the experimenter told children that one doll brushed his hair, placed the hairbrush in one cupboard in the room when he was finished and that the other doll retrieved the brush and then placed it in the other cupboard. After this, the test phase commenced where the experimenter placed each doll beside the picture of the cupboard that he had put the brush into, and children were required to decide which cupboard the brush was in now. McCormack and Hoerl (2007) were interested to see if children could make the appropriate inference about the current location of the brush based on the temporal order in which the dolls had taken their turn, even though children did not directly see the dolls take their turn in front of them.

McCormack and Hoerl (2007) found that 5-year-olds were successful at choosing the correct location, but 4-year-olds were at chance, suggesting that 4-year-olds’ ability to reason about the temporal order of the events was not as advanced as that of 5-year-olds. This finding suggests that it may be the case that young children can only perform successfully when they can see the events unfold in front of them. McCormack and Hoerl (2007) provided some evidence for this suggestion by repeating the experiment with 3-year-olds, with the apparatus set up in such a way so that children could see the events unfold in front of them. In this condition, even 3-year-olds chose the correct location. From these findings, McCormack and Hoerl (2007) argued that it is actually viewing sequences of events occur in front of them unfolding in a certain order that allows young children’s judgments to reflect the order in which events occurred, rather than their ability to represent and then reason about event order (see also McCormack and Hoerl, 2005; McColgan and McCormack, 2008; Hoerl and McCormack, 2011)

McCormack and Hoerl (2007) put forward a suggestion that may explain how young children perform successfully on reasoning tasks where they can view the sequence in front of them. They suggested that when young children (around the age of 3–4 years) view causal sequences, such as the A–E–B sequence of events, they make a causal judgment without necessarily attending to or reflecting on the entire event sequence. Young children may operate along the lines of a default: when making causal judgments, they ignore any event which occurs after the effect E. It may be the case that once children see the effect E, they may no longer encode the rest of the sequence as causally relevant. Thus, when asked causal questions they may not even consider event B in the sequence, and so will automatically choose event A as the cause. McCormack and Hoerl (2007) termed this process an encoding default process. They argued that this process is non-insightful and does not require an explicit understanding about the role of temporal order in determining the causal structure of events. It usually leads to successful performance because the temporal order in which they see the events is also the causal order. However, this account would assume that young children do not have an explicit grasp of why one event is the cause and another event is not the cause, that is, they have not grasped the significance and logical force of causal order.

McCormack and Hoerl (2007) distinguished between such an encoding default process and making temporal priority judgments by reasoning about order. They suggested that older children and adults reflect on the whole sequence, recall the order based on their memory for the event sequence, and choose the causally efficacious event based on an understanding about temporal priority and the temporal order of events. If children possess this ability, then they should never make errors when it comes to choosing the causally efficacious event providing they can remember the order in which the events happened.

Thus, in relation to the findings from the current study, it may be the case that 3-year-olds are operating along the lines of an encoding default process. As a group they are above chance in choosing the correct event as the cause because the temporal order is the same as the causal order, and because they can see the events unfolding in front of them. In order to account for developmental improvements in performance, we need to assume that this encoding process does not always function optimally (i.e., that children do not always encode A as a causally relevant event). If this is correct, 3-year-olds, when faced with the test question, will sometimes find themselves with no previously encoded information about what is causally relevant. Moreover, if they lack an explicit grasp of the temporal priority principle, they will have no basis on which to make an inference, even if they can recall the order in which the events occurred, and will have to guess. In contrast, 4-year-olds are more successful as they are able to reason about event order, putting to work the principle that causes always precede effects, which will inevitably yield the correct answer. This could be further explored by covering the entire event sequence, and then informing children afterward about the order in which the balls had been dropped. If children are basing their judgments on something like an encoding default process, then they should struggle to choose the temporally prior event if they cannot see the events unfold in front of them. By contrast, if children are reasoning about event order based on their explicit understanding that causes precede effects, then we would expect to find that children would successfully choose the temporally prior event even under these circumstances.

We have distinguished between two possible reasons for some 3-year-olds’ poorer performance: that they may have poorer memory skills or that they may lack an explicit grasp of the nature of the temporal priority principle. In the current study, we did not actually ask children to recall the order in which the events had occurred (though see Sophian and Huber, 1984), but assessing children’s memory for the event sequence along with their causal judgments may in fact be critical in distinguishing between these two explanations. If memory problems underpin children’s difficulties in the task, then we would expect to see a close relationship between the accuracy of memory for the event sequence and causal judgments, such that when children choose B as the incorrect cause they also are likely to erroneously report the order in which events occurred. Such a pattern of performance would suggest that children do understand the temporal priority principle but have difficulty putting it to work because of problems remembering event order. Alternatively, it may be that, at least for younger children, there is no close relationship between memory performance and performance on the causal task: e.g., younger children may get causal questions wrong but get memory questions correct. This second pattern of performance would suggest that children do not fully appreciate the temporal priority principle. Indeed, if young children’s causal judgments are underpinned by an encoding default process, we might expect there to be no close relationship between memory for event order and causal judgments, because the latter would not be based on the former. Rather, children’s causal judgments would reflect the extent to which they encoded each of the previous events as causally relevant at the time at which the events unfolded.

The fact that we have found development improvements in such a basic aspect of causal learning indicates that there may be important age-related changes in causal cognition that may be overlooked within the causal Bayes net approach that currently dominates developmental research in this area (Gopnik and Schulz, 2004; Gopnik et al., 2004). The Bayesian account is a computational approach that does not aim to describe the psychological processes involved in causal inference, therefore, as it stands, the issue of whether 3-year-olds’ problems stem from memory difficulties or conceptual problems is not one that Bayesian theorists need take a stance on. Indeed, Gopnik (2012) argues that even preschoolers’ causal inferences typically resemble those of an idealized Bayesian learner. Nevertheless, it could be argued that the very existence of developmental improvements in performance, such as those reported here, makes it pressing to identify what the important underlying processing changes are.

In conclusion, it has been shown that there is a difference between how 3- and 4-year-olds perform in a simple causal paradigm, with 4-year-olds performing significantly better than 3-year-olds. However, unlike findings from previous research, we found that even young 3-year-olds are unlikely to show a recency effect in their causal judgments. It is the more random performance of a sub-group of 3-year-olds that requires a developmental explanation. Our finding suggests that either that 3-year-olds’ understanding about the temporal priority principle is not as advanced as that of 4-year-olds, or that they fail to remember the order in which the events have occurred.

## Conflict of Interest Statement

The authors declare that the research was conducted in the absence of any commercial or financial relationships that could be construed as a potential conflict of interest.
